# Prognostic Signature for Human Umbilical Cord Mesenchymal Stem Cell Treatment of Ischemic Cerebral Infarction by Integrated Bioinformatic Analysis

**DOI:** 10.1155/2022/9973232

**Published:** 2022-12-13

**Authors:** Zhifeng Wen, Fangxi Liu, Xinyu Lin, Shanshan Zhong, Xiuchun Zhang, Zhike Zhou, Jukka Jolkkonen, Chang Liu, Chuansheng Zhao

**Affiliations:** ^1^Department of Neurosurgery, The First Affiliated Hospital of China Medical University, Shenyang, Liaoning Province, China; ^2^Department of Neurology, The First Hospital of China Medical University, Shenyang, Liaoning Province, China; ^3^Department of Geriatrics, The First Affiliated Hospital, China Medical University, Shenyang, China; ^4^A.I. Virtanen Institute and Institute of Clinical Medicine/Neurology, University of Eastern Finland, Finland; ^5^The Stroke Center, The First Hospital of China Medical University, Shenyang, Liaoning, China

## Abstract

In recent studies, stem cell-based therapy is a potential new approach in the treatment of stroke. The mechanism of human umbilical cord mesenchymal stem cell (hUMSC) transplantation as one of the new approaches in the treatment of ischemic stroke is still unclear. The aim of this study was to determine the traits of immune responses during stroke progression after treatment with human umbilical cord blood MSCs by bioinformatics, to predict potential prognostic biomarkers that could lead to sex differences, and to reveal potential therapeutic targets. The microarray dataset GSE78731 (mRNA profile) of middle cerebral artery occlusion (MCAO) rats was obtained from the Gene Expression Omnibus (GEO) database. First, two potentially expressed genes (DEGs) were screened using the Bioconductor R package. Ultimately, 30 specific DEGs were obtained (22 upregulated and 353 downregulated). Next, bioinformatic analysis was performed on these specific DEGs. We performed a comparison for the differentially expressed genes screened from between the hUMSC and MCAO groups. Gene Ontology enrichment and pathway enrichment analyses were then performed for annotation and visualization. Gene Ontology (GO) functional annotation analysis shows that DEGs are mainly enriched in leukocyte migration, neutrophil activation, neutrophil degranulation, the external side of plasma membrane, cytokine receptor binding, and carbohydrate binding. KEGG pathway enrichment analysis showed that the first 5 enrichment pathways were cytokine-cytokine receptor interaction, chemokine signal pathway, viral protein interaction with cytokine and cytokine receptor, cell adhesion molecules (CAMs), and phagosome. The top 10 key genes of the constructed PPI network were screened, including *Cybb*, *Ccl2*, *Cd68*, *Ptprc*, *C5ar1*, *Il-1b*, *Tlr2*, *Itgb2*, *Itgax*, and *Cd44*. In summary, hUMSC is likely to be a promising means of treating IS by immunomodulation.

## 1. Introduction

Ischemic stroke is one of the leading causes of death worldwide. Disruption of blood delivery caused by arterial obstruction reduces blood flow to the brain for nutrients, resulting in acute loss of neurons and glial cells. Impairment of the blood-brain barrier (BBB) is one of the main pathologies associated with ischemic stroke. BBB damage starts before neuronal injury occurs and influences the extent of brain damage. Limited by narrow time windows and strict indications, although intravenous thrombolysis and endovascular therapy have made great progress in the clinical management of ischemic stroke [1], patients who fail to undergo these treatments may be left with a residual deficit [2]. Current therapies have not been able to repair or replace damaged neural tissue. Recent studies have shown that stem cell therapy aims at neuroprotection of high-risk tissues during the acute phase of stroke, which may involve direct replacement of damaged brain tissues or neurorepair methods that promote endogenous repair processes in the brain, allowing for cell replacement and functional reconstruction along with repair of god-damaged neural cells, with promising applications [3]. It has been demonstrated that stem cells including embryonic stem cells (ESCs), inducible pluripotent stem cells (iPSCs), neural stem cells (NSCs), and mesenchymal stem cell (MSCs) have the beneficial effects for neurological diseases. In the treatment of ischemic stroke, MSCs are the most frequently used as seed cells, including adipose-derived MSCs, cord blood-derived MSCs, and bone marrow MSCs (BMSCs) [4]. In particular, human umbilical cord mesenchymal stem cells (hUMSCs) are noninvasive, nonhematopoietic, have no ethical issues, their access is very convenient, and they have a weak immune rejection response [5]. Although hUMSC can be an ideal stem cell type for the treatment of ischemic stroke, its therapeutic mechanism and the optimal route for transplantation are not clear.

Recently, there is growing evidence that inflammatory and immune responses after stroke are strongly associated with stroke severity and outcome [6], that immunity and inflammation play a key role in the pathogenesis of acute stroke, and differential activation or suppression of the immune system in the central or peripheral system has been considered one of the most promising targets for limiting the progression of brain damage during or after stroke [7]. Inflammation is a key progression at the onset of ischemia and after stroke and involves multiple immune cells and factors [8]. Microglia, astrocytes, and endothelial cells, which reside in the central nervous system, are involved in the immune/inflammatory activation induced by ischemic stroke [9]. Microarray studies have shown that mRNA and noncoding RNA expression profiles are extensively altered in the blood or brain after ischemic stroke [10], providing important new insights into this disease at the transcriptional and translational levels. The poststroke neuroinflammatory microenvironment is regulated by a multistage progression of factors. The main ones include resident immune cell activation, peripheral immune cell recruitment, inflammatory cytokines, and reactive oxygen species (ROS) [11].

However, scientific investigation of hUMSC is still urgently needed to deepen the understanding of stroke and to discover new therapeutic mechanisms. Bioinformatic analysis plays an important role in screening candidate biomarkers for various diseases. Recently, many researchers have confirmed the involvement of a large number of differentially expressed genes (DEGs) in hUMSC in molecular functions, cellular component, biological processes, and signal pathways [12–14]. However, uncertainties remain regarding the molecular pathways between DEGs and mRNAs in the mechanism of hUMSC for the treatment of ischemic stroke. Our study was designed to help researchers identify the underlying mechanisms between DEGs and differentially expressed mRNAs by bioinformatics. This will help to detect ischemic stroke-related biomarkers that are useful for ischemic stroke patients.

## 2. Method

### 2.1. Microarray Data

Through GEO database (http://www.ncbi.nlm.nih.gov/geo/), gene expression dataset related to hUMSC treatment of ischemic stroke was retrieved using “human umbilical cord mesenchymal stem cell and ischemic cerebral infarction” as the key words. The microarray dataset (GSE78731) consists of 16 rats' samples which based on the Agilent-028279 SurePrint G3 Rat GE8x60K Microarray (probe name version, https://www.selectscience.net/products/sureprint-g3-rat-ge-8-x-60k-microarray/?prodid=113098) platform containing extracted ipsilateral brain tissue of infarction from three groups of SD rats, respectively, the normal group (sham-operated rats, *n* = 5), hUMSC group (1 × 10^6^ hUMSC injected intravenously at 24 h after MCAO, *n* = 6), and MCAO group (saline injected intravenously at 24 h after MCAO, *n* = 5). The mRNA expression profiles of the abovementioned samples were detected based on the Affymetrix Multispecies Array platform.

### 2.2. Identification of Differentially Expressed Genes

After we obtained the data, we first used a robust multiarray averaging (RMA) algorithm, thus correcting for background and normalizing the quartile data. We excluded probes without a corresponding gene symbol before calculating the average value of the gene symbols with multiple probes. Analysis that follows was performed with R software (version 3.6.1, https://www.r-project.org/). Performing differential gene screening, taxonomic annotation, graphical plotting, and functional enrichment by using limma, clusterProfiler, dplyr, Hmisc, ggplot2, enrichplot, cowplot, topGO, pathview, http://org.Hs.eg.db, DOSE downloaded from the Bioconductor website, Student's *t*-test, and fold change (FC) were calculated for all DEGs using the limma package in R environment. We used *P* < 0.05 and |FC| > 1.5 as thresholds for significant differences in DEGs and subsequently visualized the differential genes by the ggplot2 package in R software for the analysis of volcano plots, hierarchical clustering, and DEGs.

### 2.3. Gene Function Analysis

After obtaining the gene list containing all the differentially expressed up- and downregulated genes, the data were analyzed by GO analysis using the clusterProfiler package of R software, and the species Rattus norvegicus was selected; then, the results were carried out. The horizontal coordinates of different GO terms represent the number of gene counts involved in that term. The gene count value represents the importance of the term, and the higher the count value, the greater the role the term may play. The shade of color represents the *P* value, and the smaller the *P* value, the more notable the entry of the GO term, meaning that the more likely the current result is an enrichment result rather than a random one. The GO enrichment histogram was plotted in R studio by ggplot2 package.

Using the same approach, KEGG analysis of differential genes was performed using R software to calculate the significance of differential gene enrichment for each pathway. The smaller the *P* value, the more significant the corresponding biological pathway is. Biological pathways with *P* < 0.05 were considered to be statistically significant. The higher the enrichment score, the more significant the pathway is. Specific signaling pathways were visualized using the ggplot2 package. The significant biological pathways were ranked in ascending order of *P* value.

### 2.4. Protein-Protein Interaction Network and Subnetwork

The gene symbol of the obtained differential genes was first transformed with the gene ID using the tool on the UniProt website, and the transformed ID was entered into the STRING website (https://string-db.org/) to obtain the mapped differential protein interaction network. Visualization of key gene interactions was obtained by using the cytoHubba plugin in the Cytoscape and selected PPI hub genes [15].

### 2.5. Gene Set Enrichment Analysis (GSEA)

Download the GSEA software from the website (http://software.broadinstitute.org/gsea/) and use the default parameters of the program to perform GSEA analysis. The genes in each genome can be obtained through MSigDB (Molecular Signatures Database) or publications. Leading edge analysis was performed in GSEA and visualized as heat maps using Morpheus (https://software.broadinstitute.org/morpheus/) [16].

## 3. Result

### 3.1. Identification of Differentially Expressed mRNAs

**Figure 1:**
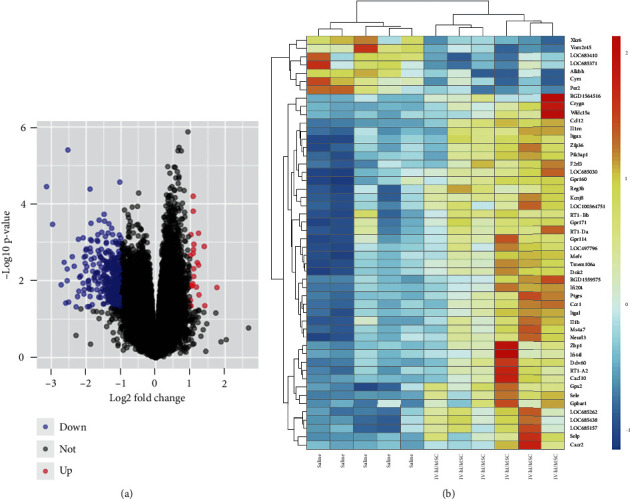
(a) Gene differential expression. Red dots represent upregulated DEGs, log_2_FC > 1.5, and adjusted *P*value < 0.05. Blue dots represent downregulated DEGs, log_2_FC < −0.5, and adjusted value < 0.05. Black dots represent |log_2_*FC*| < 1.5. DEGs: differentially expressed genes; FC: fold change. (b) Cluster heat map of DEGs. Red indicates upregulation of gene expression. Blue indicates downregulation of gene expression. White indicates no significant change in gene expression.

First, 375 DEGs (353 downregulated and 22 upregulated) were identified between the two groups, and all DEGs are shown in the volcano plot (Figure 1(a)). Subsequently, GSE78731 was clustered and divided into saline and hUMSC groups. A heat map of DEG expression is shown in Figure 1(b). In the heat map analysis, we selected the top 50 genes with the most significant differences, and based on previous studies, *Cxcr2*, *Il-1b*, *Ccr1*, and *Ccl12* can serve as predictors of the therapeutic potential of mesenchymal stromal cells. We then constructed and visualized the differential gene expression network. Images are drawn by the ggplot2 package and the heat map function in the R software.

### 3.2. Functional and Pathway Enrichment Analyses

**Figure 2:**
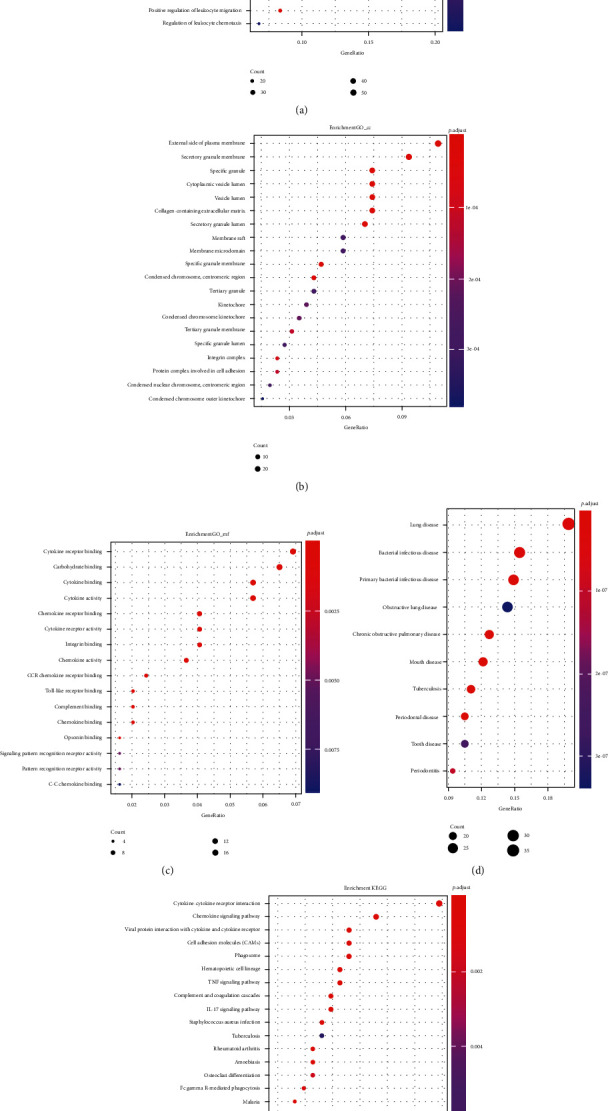
Functional annotation of DEGs. (a–c) Gene counting in biological processes (BP), cellular components (CC), and molecular functions (MF). (d) DO functional analysis. (e) KEGG pathway enrichment.

clusterProfiler package in R studio was used to explore the functions and pathways that DEGs were mainly enriched in [17]. Gene Ontology (GO) analysis included three parts: biological process (BP), cellular component (CC), and molecular function (MF) [18]. The results were considered statistically significant if *P* < 0.05. We enriched a total of 375 genes: 22 upregulated genes and 353 downregulated genes, and the results are shown in Figures 2(a)–2(d) as follows. BP analysis was mainly enriched in the following: leukocyte migration, neutrophil activation, neutrophil degranulation, neutrophil activation involved in immune response, and neutrophil-mediated immunity. These genes are mainly involved in neutrophil activation and migration, suggesting that alterations in neutrophil function may be involved in the development of ischemic stroke. The CC analysis indicated that these feature DEGs were present on the external side of plasma membrane, secretory granule membrane, etc. In addition, the enrichment results of MF indicated that the neutrophil function may be involved in the development of ischemic stroke. Also, MF enrichment results showed the cytokine receptor binding, carbohydrate binding, cytokine binding, and cytokine activity. This result corroborates the involvement of immune system alterations in stroke treatment and suggests that the application of stem cells may have a beneficial effect on the body. The application of stem cells may have a positive effect on the immune system of the body. DO analysis suggests that ischemic stroke has the most similarity with these differential genetic changes include lung disease, bacterial infectious disease, and primary bacterial infectious disease. Poststroke pneumonia may be a respiratory syndrome caused by a variety of factors. Patients with stroke often have a reduced mucus-cilia clearance system, altered mucus rheology, and a weakened cough reflex. Retained secretions can obstruct the airways, allowing bacterial colonization and causing an inflammatory response leading to airway and parenchymal damage [19]. Interestingly, these differences coincide with poststroke-associated pneumonia (SAP), and stem cell therapy for stroke may also have a positive impact on SAP. Pathway enrichment analysis of DEG was performed using clusterProfiler in the R package.  Figure 2(e) shows the top 20 most significantly different genes enriched in key signaling pathways. Five signaling pathways had higher enrichment factors than others, and they were cytokine-cytokine receptor interaction, chemokine signal pathway, viral protein interaction with cytokine and cytokine receptor, cell adhesion molecules (CAMs), and phagosome. These results suggest that inflammatory factor-mediated cytokine production and activation may be an important mechanism for MCAO development or hUMSC therapy.

### 3.3. GSEA

**Figure 3:**
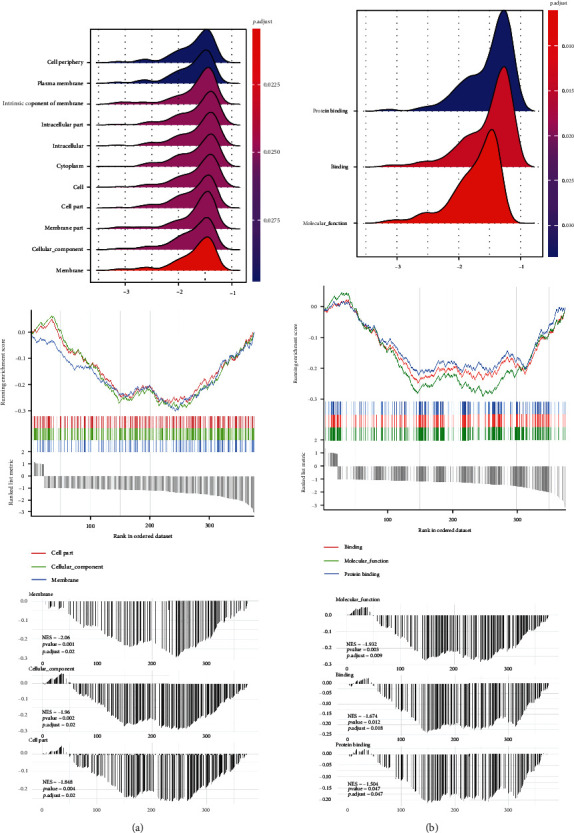
Enrichment plots from GSEA. GSEA: gene set enrichment analysis. (a) Gene set enrichment analysis in CC. (b) Gene set enrichment analysis in MF.

To explore the underlying mechanism, we performed GSEA between groups to identify the enriched KEGG pathway.  Figure 3(a) shows the results of CC analysis in SD rat samples. It can be seen that membrane, plasma membrane, intrinsic component of membrane, intracellular part intracellular, cytoplasm, cell, cell part, membrane part, cellular component, and cell periphery were all significantly enriched.  Figure 3(b) shows the results of MF analysis in rat samples. Binding, protein binding, and molecular function were significantly enriched. However, we did not find significantly enriched pathways in BP.

### 3.4. Protein-Protein Interaction Network Construction and Analysis of Modules

**Figure 4:**
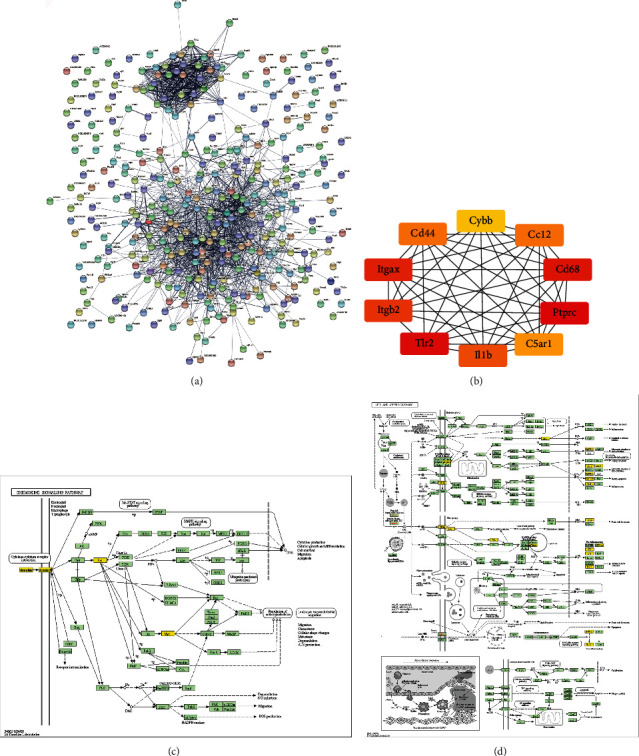
Protein interaction analysis and key gene network. (a) Protein interaction analysis of 375 genes. (b) Top 10 key genes (hub genes). (c, d) KEGG Mapper annotation of all DEGs.

Then, we used the obtained DEGs for protein interaction analysis using STRING online website database, and the obtained results are shown in Figure 4. Subsequently, we used cytoHubba in Cytoscape software to select the top key genes of the constructed PPI network and map the key gene interaction network, in which the top 10 key genes (hub genes) were *Cybb*, *Ccl2*, *Cd68*, *Ptprc*, *C5ar1*, *Il-1b*, *Tlr2*, *Itgb2*, *Itgax*, and *Cd44*, which were considered more important than other key genes due to their critical positions in the PPI network. Combined with the KEGG annotation information, we found cytokine-cytokine receptor interaction, chemokine signaling pathway, and lipid and atherosclerosis, and we suggest that cytokine-cytokine receptor interaction after cerebral infarction and downstream genes in the immune pathway may play an important influence on the formation of atherosclerosis.

### 3.5. The Network in Signaling Pathways

**Figure 5:**
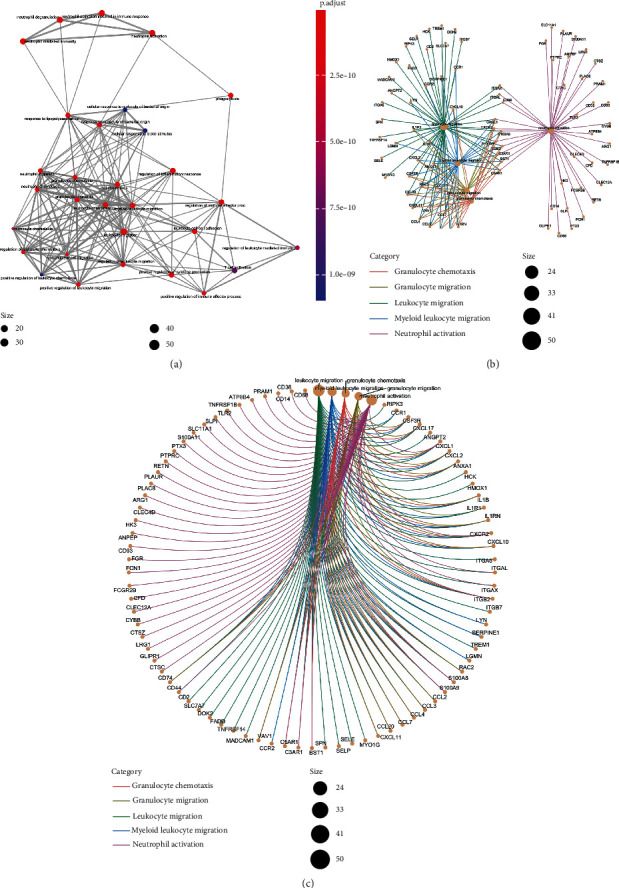
(a–c) Distribution of DEGs in different GO enrichment functions.

Figure 5(a) shows the interaction network formed after functional classification of the differential genes, and the interaction network constructed by the differential genes shows that the interactions are mainly enriched in leukocyte migration and myeloid leukocyte migration. The five major pathways are leukocyte migration, myeloid leukocyte migration, granulocyte chemotaxis, granulocyte migration, and neutrophil activation. The size of the node represents the number of genes in the pathway (Figure 5(b)), and the density of the different colored lines reflects the importance of the pathway in the total number of pathways (Figure 5(c)).

## 4. Discussion

hUMSCs may differentiate into neurons, astrocytes, or oligodendrocyte subpopulations. hUMSCs may play an active role in the treatment of ischemic brain injury through neuronal protection, neural circuit remodeling, endogenous angiogenesis, modulation of immune inflammatory responses, and regulation of neurometabolites [12, 20]. Manifested as neuroprotective, antiapoptotic, neurogenic, and angiogenic effects in neurodegenerative diseases, hUMSC can inhibit autophagy leading to loss of migration, differentiation, and antiapoptosis and promote neurogenesis and synapse formation. In contrast, hUMSCs improved motor function and promoted morphological improvement in a rat model of spinal cord injury [21]. Numerous studies have also explored the therapeutic role of hUMSCs in mediating the balance between Treg and Th17 cells. The lack of expression of immune response-related surface antigens such as *CD40*, *CD80*, and *CD86* on hUMSCs allows hUMSCs to evade host immune attack [22]. Ischemic stroke induces a profound local inflammatory response involving various types of immune cells, the important role of immune cells in the development of stroke, and the potential mechanisms of thrombotic inflammation have. The important role of immune cells in stroke development and the potential mechanisms of thrombotic inflammation have been demonstrated in animal models of ischemic stroke [23]. In ischemic stroke, the immunomodulatory role played by hUMSCs may serve as an important target to guide our clinical work.

Our study used bioinformatic analysis to screen 375 DEGs to predetermine the regulatory mechanisms of ischemic stroke. These DEGs may play a key role in the development of ischemic brain infarction treated with hUMSC. Their regulatory network may provide new insights for future studies of ischemic brain infarction mechanisms. The results of key gene analysis corroborated the results of GO and KEGG. The 10 most important key genes were *Cybb*, *Ccl2*, *Cd68*, *Ptprc*, *C5ar1*, *Il-1b*, *Tlr2*, *Itgb2*, *Itgax*, and *Cd44*. KEGG Mapper annotation results showed that they are associated with lipid and atherosclerosis. *TLR2* and *TLR4* are responsive to nerve injury, and they are upregulated in the CNS and positively correlated with the production of proinflammatory cytokines *TNF-alpha* and *IL-1* [24]. *CD45*, also known as protein tyrosine phosphatase receptor type C (PTPRC), is expressed primarily on immune cells to initiate transmembrane [25]. Some studies have found that CD45 + population homed to the site of the brain injury, decreased the lesion volume, and induced a significant beneficial effect on neurobehavioral recovery after human umbilical cord blood intravenous administration one day posttrauma [26]. After ischemia, the complement system activates a series of bioactive molecules. C5a/C5aR1 axis activation is potentially involved in the subsequent induction of tissue neovascularization. This emphasizes the role of C5a/C5aR1 in poststroke involvement in angiogenesis [27]. And *C5ar1* antagonists also have neuroprotective effects [28].

Stroke-associated pneumonia (SAP) is a common medical complication of stroke that affects [29]. Acute ischemic stroke triggers can cause severe systemic and local immune dysfunction, increasing the susceptibility of the body to infections that can result in stroke-associated pneumonia. hUMSCs can decrease the level of inflammatory cytokines and increase the expression of anti-inflammatory cytokines, and coadministration of hUMSCs and LZD (Linezolid) reduces lung inflammation more significantly [30]. The DO analysis showed that the genes were enriched in lung disease and bacterial infectious disease, suggesting that the treatment of our hUMSCs may have altered both the regulatory factors in the SAP-related pathway and become one of the favorable factors for the control of poststroke infection. However, this is only inference, and further experimental studies are still needed to verify the results of these bioinformatic analyses.

The interaction between platelets and leukocytes is particularly associated with secondary stroke and contributes to plaque formation and arterial stenosis [31]. Similarly, it was demonstrated that hUMSCs significantly prolong the survival and function of human neutrophils through the combined action of *IL-6*, *IFN-b*, and granulocyte macrophage colony-stimulating factor. This was similarly demonstrated in our GO analysis and in the pathway interaction network, and signal pathway interactions were focused on leukocyte migration, neutrophil activation, and neutrophil degranulation. The lipid and atherosclerosis pathway was also one of the major enriched pathways in the results. However, the question of how hUMSCs interfered with leukocyte interactions and their impact on the inflammatory and thrombogenic processes occurring in the context of stroke remains one of the directions for future research.

In summary, we hypothesize that treatment with stem cells can cause changes in peripheral immunity by causing alterations in key genes, which in turn affect protein synthesis in peripheral blood and central immune cells. Given the low immunogenicity of human bone marrow mesenchymal stem cells and the ease of obtaining large numbers of cells by in vitro expansion, they have a promising clinical application.

## Data Availability

Microarray datasets, GSE78731 (mRNA profile), of middle cerebral artery occlusion (MCAO) rats were obtained from the Gene Expression Omnibus (GEO) database (https://www.ncbi.nlm.nih.gov/geo/). The data contains the original images and the original files, and the raw data are available from the corresponding authors (CZ: cszhao@cmu.edu.cn). Permission to reuse the data needs to be granted with the permission of the journal and the corresponding authors.
